# Learner’s Stop the Bleed Outcomes Between Lay Instructor and Emergency Medical Services (EMS)-Trained Instructor Groups

**DOI:** 10.7759/cureus.45846

**Published:** 2023-09-24

**Authors:** Jeffrey L Pellegrino, Stephen E Smith, Abigail Nolan, Nathan Charlton, Craig Goolsby

**Affiliations:** 1 Emergency Management and Homeland Security, The University of Akron, Akron, USA; 2 Emergency Management, Aultman Hospital, Canton, USA; 3 Emergency Medicine, University of Virginia, Charlottesville, USA; 4 Emergency Medicine, Harbor-University of California Los Angeles (UCLA) Medical Center, Torrance, USA

**Keywords:** hemorrhage control, tourniquet, instructor, american red cross, life-threatening bleeding, first aid, stop the bleed™

## Abstract

Background: One of the most utilized Stop the Bleed courses, the “Bleeding Control Basic (BCon) course v. 1.0,” requires instructors to have a specific healthcare license or pre-hospital credential (e.g., physician or paramedic) or specific emergency medical services (EMS) instructor certification and have completed the BCon provider course. This requirement provides a level of expertise in instructors but limits the potential workforce for sharing life-saving knowledge and skills. Other Stop the Bleed courses, such as the American Red Cross First Aid for Severe Trauma (FAST) course, do not have this requirement. This raises questions pertaining to the learners' outcomes between those facilitated by instructors with and without healthcare licenses or credentials.

Methods: Learners' outcomes for applying a tourniquet (skill), knowledge (cognitive), and Intention to Aid (attitude for behavior) were compared between those taught by lay instructors and EMS-trained (emergency medical technician or paramedic) instructors. All were trained as new instructors in the FAST program.

Results: For the study’s primary outcome, all of the learners (n=135) properly applied a tourniquet to a simulated leg injury at the end of the training based on video evidence (skill). Learners in the EMS-trained instructor groups (n=84, mean age 25.5 years, 68% female), who were older and had more education, scored significantly higher on knowledge of tourniquet use on the Stop the Bleed Educational Assessment Tool (SBEAT) (mean=90.0 vs. 83.9 on a scale of 0-100, p=0.001) with a small effect size than the lay instructor group (n=51, mean age 16.6 years, 88% female). There was no statistical difference in attitude toward helping behaviors in a bleeding emergency between the two groups on the Intention to Aid (I2A) survey.

Implications: Lay instructors and EMS-trained instructors performed comparably in facilitating a widely available Red Cross Stop the Bleed course. Lay experience with tourniquets should not disqualify individuals from being a Stop the Bleed instructor. Using a standard curriculum with instructor development offers a way for people with and without an EMS background to teach life-saving competencies effectively.

## Introduction

Accidents in the home, in traffic, and in the workplace are common occurrences that many people will experience at some point in their lives, with the potential to cause life-threatening bleeding; they also then have the opportunity to take life-saving action [1,2]. As the leading cause of death from traumatic injury is blood loss, early recognition, appropriate intervention, and activation of the emergency medical system, are needed to reduce sequelae and mortality. The principles of Stop the Bleed (STB) offer individuals and populations a means to render care but require an appropriate educational approach and program accessibility for effective implementation [3,4].

In-person training for lay responders in the control of life-threatening hemorrhage is an efficacious manner for learning tourniquet application skills by the end of a course [5]. To scale a public health campaign, like STB using in-person instruction, more people are needed to facilitate training in more locations for more populations [4,6]. Currently, the STB “Bleeding Control Basic (BCon) course v. 1.0” requires instructors to have a specific healthcare provider (e.g., EMT, RN, MD) or instructor certification (e.g., National Association of Emergency Medical Technicians {NAEMT}, Advanced Trauma Life Support {ATLS}, Tactical Combat Casualty Care {TCCC}/Tactical Emergency Casualty Care Committee {TECCC}) and completed the Bleeding Control (BCon) course [7].

Teachers with a clinical health background do not always have superior outcomes to non-clinical teachers raising the question of the value added by emergency medical services (EMS) backgrounds on learner outcomes [8]. Multiple studies have also demonstrated the efficacy of medical students to serve as instructors, once completing the basic course [9-13]. In 2020, we developed our research question to evaluate the differences between instructors with a clinical healthcare (e.g., EMS/ medical) background compared to layperson instructors on learners' outcomes. Specifically, the primary outcome of interest was the ability to apply tourniquets successfully after instruction. In addition, we aimed to evaluate if there are differences in knowledge and intention to help between groups. We hypothesized that there would be no difference in learner outcomes of placing a tourniquet on a simulated injury. The University of Akron’s Institutional Review Board provided ethical oversight and approval for this research (#20210601).

## Materials and methods

We used a prospective experimental methodology in a field-based context of STB learner outcomes between courses facilitated by an EMS-trained instructor and lay instructors. Three assessment tools provided measurements to triangulate findings to identify differences in learning outcomes.

Assessment tools

Instructor participants wore a video camera on their head or chest to video record their point of view as the primary source of evidence for assessing tourniquet application. Videos were then downloaded for assessment by research team members blinded to the instructor participant group status. Evaluators assigned “successful application of a tourniquet” when an individual placed the tourniquet 2-3 inches from the simulated wound and a ¼-inch diameter piece of doweling could not be slipped between the training device and tourniquet, and the windlass (twisting device) was secured. The training device consisted of a simulated upper leg that measured 15 inches in length by 20 inches in circumference (compressible to 19 inches with a tightened tourniquet) with an “injury” located toward one end to allow proximal tourniquet placement.

Two additional tools were used to quantify the learning outcomes of the course participants. The Stop the Bleed Educational Assessment Tool (SBEAT) provides a standard measure of personal knowledge of life-threatening bleeding and the competencies to control it by lay responders [14]. The SBEAT uses 14 questions to ascertain learners’ recall and ability to apply content along a continuum of difficulty. For example, easy questions show pictures of various bleeding from which the learners identify life-threatening examples. A more difficult question asks if a tourniquet is appropriate to stop life-threatening bleeding from the hand. The SBEAT utilizes a Rasch analysis to measure latent traits of tourniquet competencies, showing the probability of an individual getting a correct response on a test item or “Person measure,” while allowing for statistical analysis after the transformation of ordinal data.

Additionally, the Intention to Aid tool (I2A) [15] measures the affective domain of helping behaviors by applying the Theory of Planned Behavior to better understand lay responders’ intent to use life-saving skills [16]. Consisting of 30 Likert prompts, the survey is based on the Theory of Planned Behavior’s domains of personal attitudes, social norms, and confidence, as well as self-identified intention based on the situation. These results were also analyzed in a Rasch manner to provide reliable Person outcomes for statistical analysis. Both the SBEAT and I2A were collected via Qualtrics after the course finished. All Rasch analyses were conducted in Winsteps and statistical analyses were conducted in Microsoft Excel (Office 365) [17].

Participants

We prospectively recruited, trained, and resourced EMS-trained and lay instructor participants to facilitate the American Red Cross First Aid for Severe Trauma (FAST) course from the Akron, Ohio region and the Charlottesville, Virginia region. Sixteen individuals volunteered to take part in this study. All instructor participants were informed of the goals of the study and consented to participate but were blinded to characteristics of interest. Twelve individuals completed the 8-hour FAST instructor course and the study orientation. Eight instructors submitted data from their courses.

In the EMS-trained instructor group (n=6), two were paramedics and four were EMT-certified providers. Both paramedics had field experience with placing tourniquets. In the lay (n=2) instructor group, the two people had prior first aid courses with a tourniquet component. No instructor participants were previously trained as Red Cross instructors. Each instructor participant facilitated up to three courses in the community or schools using the synchronous (135 minutes) or hybrid skill session (45 minutes) (instructor-led) portion of the program. Instructor participants facilitated eight courses in high school settings, six being hybrid; and 10 courses in the community (non-EMS or medical audiences), five being hybrid.

Course participants

All course participants were informed of their opportunity to contribute to the research and consent was obtained before the start of each course. Data collection began in October 2021 and ended in April 2022. Raw survey data were collected from 177 course participants, individuals who did not complete the assessments were removed, leaving 135 cases for analysis. There were 84 learners educated by EMS-trained instructors and 51 learners in the lay instructor group. Table 1 summarizes their pertinent demographics. Fisher's exact tests were run on binary data and a t-test for age. Groups were significantly different in demographics except for experience with tourniquets. The lay instructors worked with younger, less educated, and those less experienced in first aid. We describe and consider the potential influence of these differences in the Discussion section on the outcomes.

**Table 1:** Demographics of groups of interest (n=135). EMS: emergency medical services

Variables	EMS instructor group (n=84)		Lay instructor group (n=51)		p-Value
	n	%	n	%	
Identified as female	57	67.9%	45	88.2%	0.008
Average age (years)	25.5	-	16.6	-	0.001
Bachelor’s degree or higher	15	17.9%	0	0%	0.001
Prior first aid training	62	73.8%	25	49.0%	0.005
Previous tourniquet training	35	41.7%	14	27.5%	0.102
Previous tourniquet practice	23	27.3%	8	15.7%	0.139

Collected content data were analyzed using the polytomous Rasch model to convert Likert rating scale data into ratio data (logits) which then were appropriate for parametric analyses. Rasch analysis of the data was done in one group with fit statistics generated to provide the level of detail that can be associated with the individual measures. The SBEAT Item-Outfit mean square (MNSQ) was 0.98, which is interpreted as a reasonable fit lying between 0.5 and 1.5, and Person separation was 0.24 [18]. An item separation index value of 1.5 is required for analysis at the individual level and 2.5 is required for analysis of groups, the SBEAT was 2.88. The table in the appendix shows the reliability, separation, item fit statistics, and point-measure correlation coefficients. The I2A Item-Outfit MNSQ was 0.81 and acceptable. Person separation was 1.85, which is greater than the 1.5 threshold. Item separation was 4.85 allowing for individual and group analysis. We conducted Mann-Whitney U tests on the Person measures of the SBEAT and I2A independently. SBEAT and I2A Person scores were transformed to a range from 0-100 and must not be associated with percent correct. Prior to analysis between groups, distributions were checked for similarity and then Mann-Whitney U tests were run, using an alpha level of 0.05 as the level of significance.

## Results

**Figure 1:**
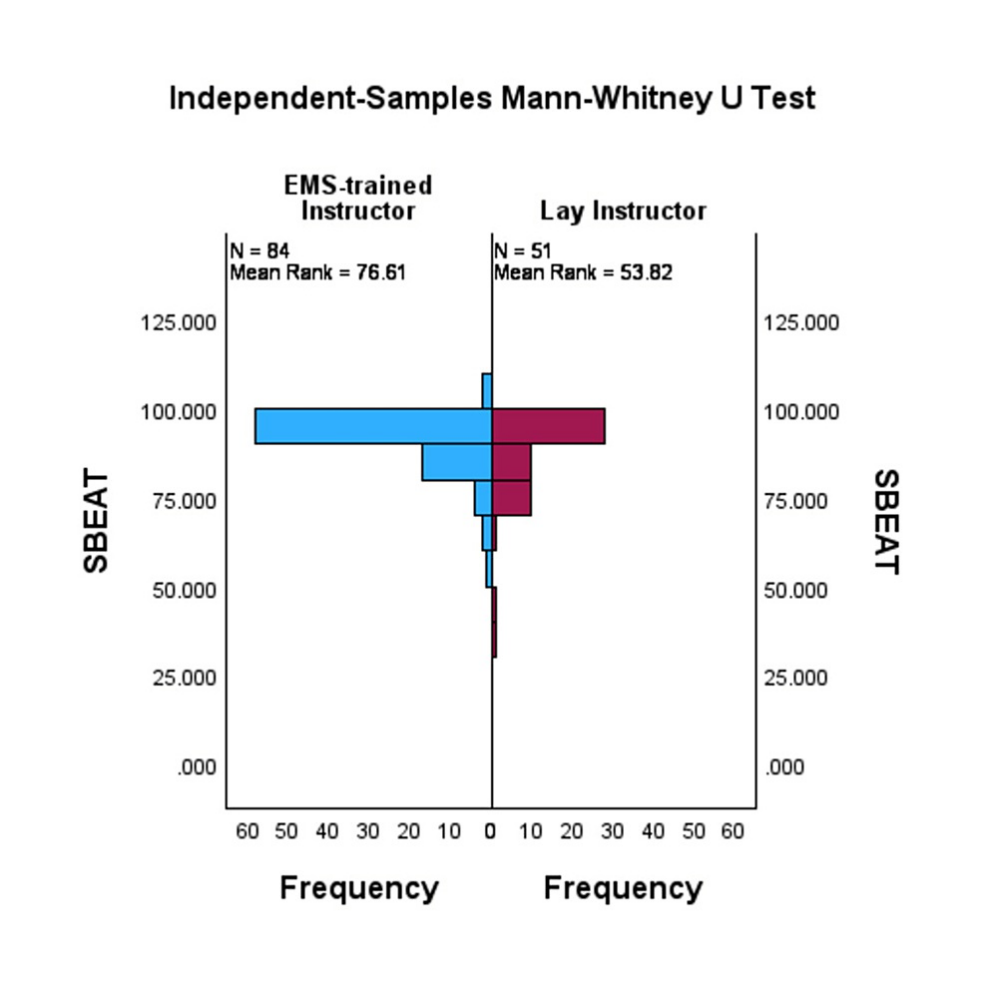
Bi-histogram comparing the distribution of the EMS-trained instructor group (left) with the distribution of the lay instructor group's (right) SBEAT scores. SBEAT: Stop the Bleed Education Assessment Tool Cognitive: 0 (low)-100 (high)

For the primary outcome, three research team members, blinded to the variable of clinical background, independently reviewed 15 courses videos for the correct placement of a tourniquet (location and tightness) at the end of the course. All course participants (100%) for groups facilitated by EMS-trained and lay instructors successfully placed a tourniquet by the end of the course. A Mann-Whitney U test showed that the SBEAT score was significantly greater for the EMS-trained instructor group (median=91.55) than the lay instructor group (median=90.00), U=1419.0, p≤0.001. Figure 1 shows the frequency distribution of SBEAT. The effect size was small with an r=-0.29.

**Figure 2:**
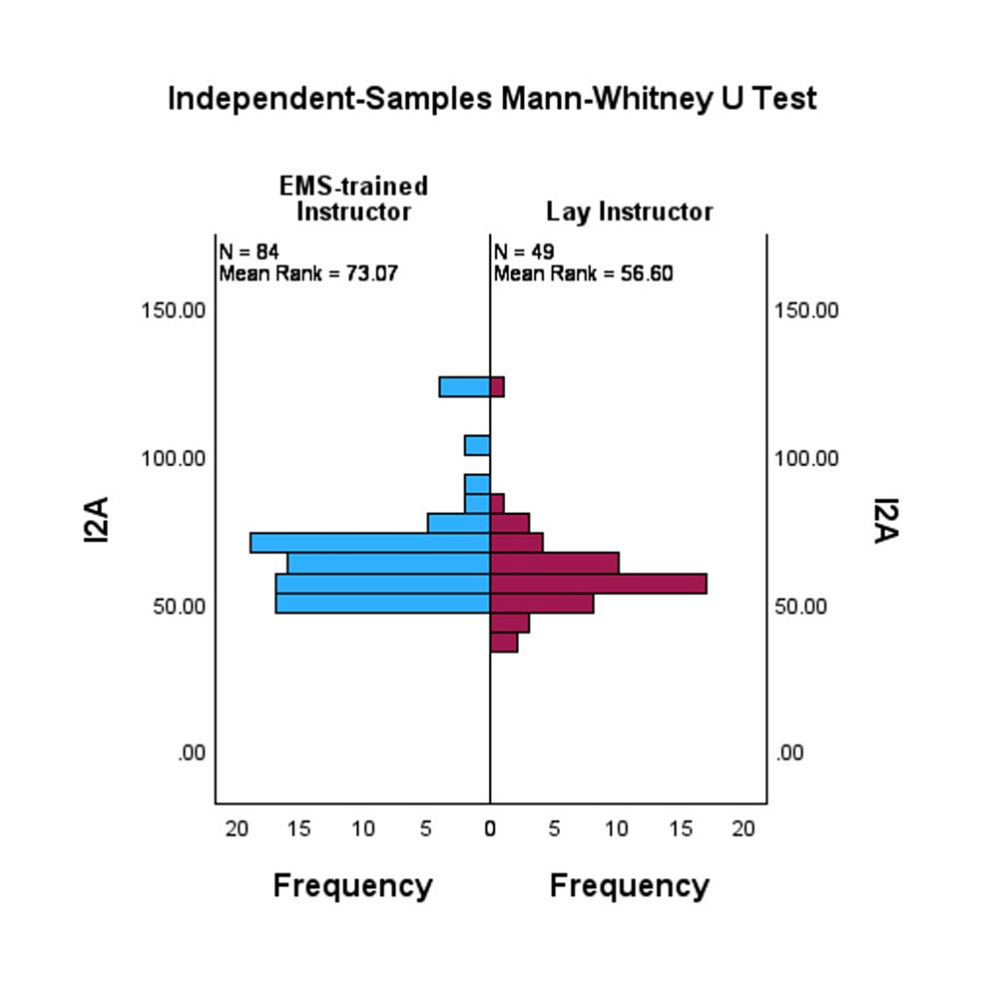
Bi-histogram comparing the distribution of the EMS-trained instructor group (left) with the distribution of the lay instructor group's (right) I2A scores. I2A: Intention to Aid scale Affective: 0 (low)-100 (high)

A Mann-Whitney test indicated that the I2A scores were statistically different between the EMS instructor-led (median=63.77) and the non-EMS instructor-led learners group (median=58.71), U=1548.5, p=0.02. The effect size was small with an r=-0.21. Figure 2 shows the frequency distribution of I2A.

A Pearson correlation coefficient was computed to determine the relationship between the SBEAT and I2A scores. The results indicate a significant and weak relationship between the two (r{135}=0.193, p=0.026). Further analysis identified the correlation between various demographics and the outcomes of interest. Gender was examined with an SBEAT Eta value of 0.199 and an I2A Eta value of 0.063, which represent approximately 4% and <1% of the variance being accounted for, respectively. Spearman's rank correlations were computed to assess the relationship between the SBEAT or I2A scores and age, education levels, and the number of previous first aid trainings (Table 2).

**Table 2:** Correlation outcomes of participant characteristics to SBEAT and I2A scores. SBEAT: Stop the Bleed Educational Assessment Tool (cognitive); I2A: Intention to Aid (affective)

Variables	SBEAT		I2A	
Demographic	r (133)	p-Value	r (131)	p-Value
Age	0.232	0.007	0.029	0.074
Educational level	0.338	<0.001	0.084	0.338
Count of previous first-aid training	-0.049	0.574	0.074	0.4

## Discussion

The primary outcome of placing a tourniquet and tightening it appropriately did not differ from courses taught by EMS-trained instructors and lay instructors. The 100% success of tourniquet placement as an outcome is higher than some reported outcomes [19]. Several considerations should be taken with this outcome as the participants were not formally tested after the class but rather achieved the outcome of placing the tourniquet having been given a scenario in which they manage scene safety, calling for help/resources, apply pressure, apply a tourniquet, and then position the victim. Also, those who struggled were given multiple opportunities for success before the “Putting It All Together” section. This last section is immediately preceded by a skill practice that has participants coaching, supporting, and participating in the practice up to four times. The outcomes of this study were similar to other full course studies that observed placement at the end of the course [10,20].

To receive the FAST training certificate, in either the hybrid or instructor-led course, each learner needed to complete a standardized “knowledge check” at the end of each lesson. Knowledge checks correlated with the FAST course outcomes of - (1) state the importance of personal safety for the responder and for the injured person; (2) distinguish life-threatening bleeding from non-life-threatening bleeding, demonstrate high-level communication skills in an emergency setting; (3) describe methods used to stop bleeding; and (4) describe positions of comfort for an injured person. All learners received a FAST training certificate representing a basic knowledge assessment.

A final exam consisting of 15 questions is used in the hybrid course and is available for pedagogical purposes but completion of it is not required in the instructor-led course. A score of 80% or better is considered a passing score. As all of the learners completed the course requirements, we could not distinguish the quality difference between the instructor groups on these knowledge elements. The SBEAT purposefully discriminated learners along a continuum and is not considered a “test” of competence at any specific mark (e.g., 80%), but rather the distribution of the participants. The EMS-trained instructor group scored significantly higher on the mean on the SBEAT (90.0 vs. 83.9). The SBEAT questions that helped discriminate these groups were identifying anatomical areas appropriate and inappropriate for tourniquets and knowing what step to take, from a given list, if the initial tourniquet does not stop the bleeding. As noted above, the demographics were different between groups, interestingly there was low correlation between gender, age, and educational levels. Interestingly, the number of previous first-aid courses didn't correlate (non-significant) with the SBEAT score. This leaves the opportunity to further research the qualities of EMS-trained instructors in knowing and developing tourniquet competencies in others so that lay instructors could use similar approaches in the future.

As reported in the Results section, the effect size was small to very small demonstrating a weak relationship between the instructor backgrounds of the groups to their scores. Knowledge of life-threatening and non-life-threatening bleeding, placement on extremities, not removing a placed tourniquet, and other foundational knowledge was known to the majority in both groups. Orlas et al., in a randomized controlled study between physicians and medical students, found learning success by all instructors, even those with less experience but with adequate training [21].

The similarity in the I2A scores between groups (66.1 vs. 59.6, p=0.14) demonstrates that the standardized curriculum doesn’t discriminate between learners regarding intentions to help. The personal attitude domain showed some hesitance in acting if they thought they might be sued. There was also a lower number of people in each group who felt that others were expecting them to act in an emergency, which may show an opportunity to improve the development of pro-action social norms. In the confidence domain, both groups reported high confidence and intention to help across the chain of survival behaviors, given a life-threatening bleeding scenario, including calling for help and providing care [22]. Demographically, there were only low to very low correlations to the I2A score.

Strengths

One of the goals of this study was to utilize “real world” or field-based courses and participants, which for the Red Cross includes school-based and community populations. We also included full instructor-led and hybrid courses in both groups. To reduce the conflation of multiple STB courses and be able to assess the experience and not the curriculum, every instructor participant completed the 8-hour FAST instructor’s course, released in 2021, which included teaching practice. All had access to an instructor’s kit consisting of course handouts, four tourniquets, a bleeding limb simulator, four dressings, a measuring dowel, and an action camera with a head/chest strap.

Limitations

A major limitation may be construed in the statistical difference in demographics of the FAST participants between the instructor groups. The combination of maturity and education in the EMS-trained instructor group may account for parts of the SBEAT knowledge gap. A larger sample is needed to determine the influence and distinction of education and first aid training experience.

Due to the circumstances of the coronavirus disease 2019 (COVID-19) on the social interaction and personal health of participants, the pool of instructors who were able to collect data became limited. COVID-19 and its sequelae also led to multiple courses being canceled after being scheduled, which limited data collection. More data would be needed to look at and between course delivery methods: face-to-face vs. hybrid. Three video recordings didn’t occur due to technical errors during collection.

The I2A outcomes were high within this sample of FAST course participants, which is likely due to the course participants being interested in the topic and willing to participate vs. a random sample from the population eligible to participate in the course. This was a methodological decision based on our wish to have a field test of those who would take the course and not random individuals. Future field research might include a comparison of a structured curriculum vs. observation of a non-standardized curriculum or pedagogy to elicit differences between educators, as well as pre-post comparisons. Also, there is evidence that skills and willingness go down within weeks and months, which should be another area of research between instructor groups [5,23].

## Conclusions

In a prospective field study (n=135) to identify differences in learners' outcomes, there was 100% success in being able to apply a tourniquet correctly to a simulated wound by the end of the course between groups taught by lay and EMS-trained instructors. Quality differences were identified in the technical/knowledge competencies, using the SBEAT, favoring the EMS-experienced instructors, with a small effect size. The intention to help in a bleeding emergency; however, was not significantly different between groups. We can conclude from this field-based study that lay instructors and EMS-trained instructors can effectively facilitate a given STB curriculum after instructor training but the factors regarding the qualities that EMS-trained instructors bring to the training should be further examined. Future research on the longitudinal retention of skills, knowledge, and Intention to Aid may differentiate outcomes of learners with different backgrounds. The proliferation of Stop the Bleed education as a public health campaign would benefit from the training and support of lay trainers/instructors/educators.

## Appendices

**Table 3:** SBEAT - reliability, separation, item fit statistics, and point-measure correlation coefficients. MNSQ: mean square; ZSTD: z-standardized; CORR: correlation; PT-measure: point-measure; P. SD: population standard deviation; SBEAT: Stop the Bleed Educational Assessment Tool

No.	Item	Infit			Outfit			PT-measure		
		MNSQ	ZSTD	MNSQ		ZSTD			CORR	
Q86_1	Which of the following needs immediate first aid to stop the bleeding? - Dripping blood from a cut	0.965	-0.179	0.715		-0.979			0.499	
Q86_2	Which of the following needs immediate first aid to stop the bleeding? - Gushing blood that will not stop coming out, even with a hand pushing on it	Minimum measure (i.e., no one missed it)								
Q86_3	Which of the following needs immediate first aid to stop the bleeding? - Flowing blood from an injury that you are pressing on	Minimum measure								
Q86_4	Which of the following needs immediate first aid to stop the bleeding? - Blood spotting through several layers of cloth	1.047	0.511		1.916		3.162			0.375
Q90_1	A tourniquet can be applied to stop an LTB from which of the following areas? - Leg	1.011	0.231		0.694		-0.019			0.178
Q90_2	A tourniquet can be applied to stop an LTB from which of the following areas? - Arm	0.894	-0.049		0.479		-0.410			0.283
Q90_3	A tourniquet can be applied to stop an LTB from which of the following areas? - Hand	1.250	2.351		2.229		3.702			0.204
Q90_4	A tourniquet can be applied to stop an LTB from which of the following areas? - Stomach	1.107	0.371		1.338		0.641			0.146
Q92	If you are alone with somebody who is not awake and has LTB coming from an arm/leg, what best describes your first action(s)?	1.003	0.091		1.454		0.992			0.349
Q94	Put the tourniquet above the wound so that it is between the torso/ body and the wound.	1.082	0.611		1.042		0.241			0.407
Q96	After placing the windlass tourniquet above the injury, it is critically important to tighten it as...	0.834	-0.399		0.647		-0.259			0.371
Q98	If the person is in pain, the tourniquet is on too tight.	0.961	-0.019		0.652		-0.329			0.320
Q100	You have applied a tourniquet to an LTB on a leg. The bleeding appeared to stop initially but has now restarted. Which of the following should be the next step...	0.724	-2.809		0.570		-2.839			0.677
Q102	When should a tourniquet be removed?	1.032	0.211		0.719		-0.239			0.337
Q114	You have applied a tourniquet to stop life-threatening bleeding. When can you loosen the tourniquet?	0.726	-1.019		0.260		-1.540			0.517
Means		0.973	0.079		0.98		0.259			-
P. SD		0.14	1.1		0.57		1.7			-
Reliability of item difficulty measures		0.89								
Item separation		2.88								
Reliability of Person ability		0.89								
Person separation		0.57								
Cronbach Alpha (KR-20) "test" reliability		0.57								
